# Cost-of-illness of cholera to households and health facilities in rural Malawi

**DOI:** 10.1371/journal.pone.0185041

**Published:** 2017-09-21

**Authors:** Patrick G. Ilboudo, Xiao Xian Huang, Bagrey Ngwira, Abel Mwanyungwe, Vittal Mogasale, Martin A. Mengel, Philippe Cavailler, Bradford D. Gessner, Jean-Bernard Le Gargasson

**Affiliations:** 1 Agence de Médecine Préventive, Abidjan, Côte d’Ivoire; 2 Agence de Médecine Préventive, Ferney-Voltaire, France; 3 The Polytechnic, Blantyre, Malawi; 4 International Vaccine Institute, Seoul, Republic of Korea; 5 Agence de Médecine Préventive, Paris, France; Mahidol-Oxford Tropical Medicine Research Unit, THAILAND

## Abstract

Cholera remains an important public health problem in many low- and middle-income countries. Vaccination has been recommended as a possible intervention for the prevention and control of cholera. Evidence, especially data on disease burden, cost-of-illness, delivery costs and cost-effectiveness to support a wider use of vaccine is still weak. This study aims at estimating the cost-of-illness of cholera to households and health facilities in Machinga and Zomba Districts, Malawi. A cross-sectional study using retrospectively collected cost data was undertaken in this investigation. One hundred patients were purposefully selected for the assessment of the household cost-of-illness and four cholera treatment centres and one health facility were selected for the assessment conducted in health facilities. Data collected for the assessment in households included direct and indirect costs borne by cholera patients and their families while only direct costs were considered for the assessment conducted in health facilities. Whenever possible, descriptive and regression analysis were used to assess difference in mean costs between groups of patients. The average costs to patients’ households and health facilities for treating an episode of cholera amounted to US$65.6 and US$59.7 in 2016 for households and health facilities, respectively equivalent to international dollars (I$) 249.9 and 227.5 the same year. Costs incurred in treating a cholera episode were proportional to duration of hospital stay. Moreover, 52% of households used coping strategies to compensate for direct and indirect costs imposed by the disease. Both households and health facilities could avert significant treatment expenditures through a broader use of pre-emptive cholera vaccination. These findings have direct policy implications regarding priority investments for the prevention and control of cholera.

## Introduction

Cholera remains an important public health problem in many low- and middle-income countries. Cholera can be prevented simply through provision of safe water and adequate hygiene and sanitation. [1] Because of this, urgent calls have been made at international level to improve health and living conditions, particularly in developing countries, through the millennium development goals [2] and more recently through the adoption of the sustainable development goals (SDGs). [3] Notable among the SDGs is the improvement of people’s access to improved water and sanitation through objectives 6.1 and 6.2 [3] whose ultimate aim is to reduce the burden of diarrheal diseases, including cholera. Despite this improvement, it is estimated that 2.9 million cases of cholera continue to occur worldwide every year with an estimated yearly mortality of 95,000. [4] Children under five year’s old [5] and sub-Saharan Africa bear most of the human and economic burden of cholera. [4,6–8]

Recurrent cholera outbreaks are reported worldwide, especially from endemic countries. [9,10] Consequently, efforts have been made to implement large control programs aimed at mitigating the consequences of cholera for individuals, families and societies. Strategies adopted for cholera prevention include promotion of hygiene, access to safe water and sanitation, appropriate case management and vaccination. [11] Several studies have documented the feasibility of mass vaccination campaigns in various places [12–15] and their protective effect. [13,16,17]

Shanchol, a killed whole-cell oral cholera vaccine (OCV) has been pre-qualified by the World Health Organization for use in cholera prevention. [18] The price of a double dose of this vaccine is currently around US$3.7, [8] with vaccine delivery costs ranging from US$1.14 (I$4.08) to US$3.05 (I$14.39) per fully immunized person in 2014 dollars. [19] However, evidence to support its widespread use in prevention of cholera is limited. Particularly, additional quality data on the burden of cholera, costs-of-illness, delivery costs and cost-effectiveness of vaccines are needed to support evidence-based decisions regarding the use of OCV in the prevention of cholera in sub-Saharan countries.

Historically, cholera has been endemic in Malawi. [20] In March 2016, a cholera vaccination campaign was implemented in Lake Chilwa area in response to a massive cholera outbreak. However, so far no study has investigated the cost-of-illness from cholera in Malawi. The aim of cost-of-illness studies is to itemize, value, and sum the costs of a health problem with the objective to estimate its economic burden. [21,22] The purpose of this study is to investigate the cost-of-illness of cholera to households and health facilities in Machinga and Zomba Districts.

## Methods

### Ethics statement

This study was approved by the National Health Sciences Research Committee (NHSRC) of the Ministry of Health in Malawi (approval number: NHSRC # 16/3/1559). All participants provided written informed consent prior to interviews. All data were handled confidentially and made anonymous before analyses.

### Study sites and participants

This study was conducted in Machinga and Zomba, two of the 28 administrative districts of Malawi. The two districts, surrounding the Lake Chilwa, are bordering Lake Chilwa and located in the southern part of the country. They had an estimated total population of 1,238,592 inhabitants in 2014. [23] Machinga and Zomba districts’ economies are based largely on agriculture, with maize as the main food crop and tobacco as the main cash crop. Fishing on Lake Chilwa is of particular importance as a source of income for the local lake bordering communities. Health services are mainly delivered through health posts, clinics, health centres and hospital facilities. In addition, many people get medical treatment from traditional practitioners and birth attendants. Moreover, people living on and around the Lake Chilwa use the lake as a source of drinking water and for their ablutions. Levels of fecal contamination are high, particularly in parts of the lake with stagnant water. [20] In consequence, the mortality attributable to exposure to unsafe water, sanitation and hygiene condition is high, at 26.1 per 100,000 inhabitants in 2012. [24] Respectively, 59% and 10% of the total Malawian population has no sustainable access to improved sanitation and drinking water sources, with large disparities among the country’s regions, which means a large proportion of the population is at high-risk for cholera. [24]

### Costs data collection aspects

We conducted a cross-sectional study using cost data collected retrospectively to estimate the cost-of-illness of cholera for households and health facilities. All the data were collected between February and March 2016 in parallel with the implementation of a mass oral vaccination campaign against cholera (February 16 to 22, 2016 and from March 8 to 15, 2016, for rounds 1 and 2, respectively). Expenses for an individual patient and for the health system were only counted once and did not include multiple disease episodes. Data from this study could be accessed through a request to the Agence de Médecine Préventive–AMP: website http://amp-vaccinology.org/fr/contact-amp.

### Measuring household cost of cholera

Only cholera cases reported from the last 7+ weeks (i.e. between February 4^th^ and March 26^th^, 2016) preceding the data collection period were eligible for interviews. A total of 100 patients were purposefully recruited for the household cost-of-illness. Eligible patients were cases presenting with an infectious disease characterized by intense vomiting and profuse watery diarrhea, regardless of vaccination status, that received care over the previous five weeks. The sample included approximately 9% of the reported cases in the study. For patient recruitment, we systematically selected every fifth patient out of those who received treatment during the five weeks preceding the survey. This was achieved by reviewing line lists–which recorded patient details in a consistent manner–located at cholera treatment facilities in the two districts. All selected patients were then visited in the community with the support of community-based health surveillance assistants, for face-to-face interviews in their homes to elicit costs incurred in seeking care. Interviews were conducted with the head of household, and/or the main patients’ caregiver who was most familiar with the costs borne during the cholera episode.

Five specifically trained interviewers collected data from all confirmed cholera patients by means of a pilot-tested structured questionnaire programmed on hand held tablets. Prior to fieldwork, interviewers were given comprehensive training on data collection procedures. We asked questions related to patients’ demographic characteristics, disease duration, health expenditures including direct medical expenses on medicines, and consumables, direct non-medical costs including transportation, foods and beverages and other miscellaneous expenses. Data relating to treatment outcome, coping strategies used by households to compensate for direct and indirect costs were also collected. Moreover, we asked currently employed persons for the estimated number of days absent from work and related self-reported total income lost. For deceased patients, we estimated lost income by asking the patients’ relatives to describe income lost during the patient’s period of illness. Intangible costs (e.g., costs of stigma from cholera), costs following death, and school days missed were not collected. Likewise, expenses relating to diagnostics and hospitalization were not considered in the household cost measurement as they were not supported by patients but covered by the Government. The household study questionnaire is given in the supporting document **S1 Appendix**.

### Measuring health provider cost of treating cholera

Facility-based surveys were undertaken in order to assess the costs to health providers for treating cholera cases. Four cholera treatment centres and one district hospital expected to manage cases were selected for data collection. Cost data collected included staff, medicines and consumables. Unit costs of medical services used to estimate the costs of treating cholera were directly measured from the study facility by standard methods. [25] A micro-costing bottom-up approach was used to estimate the amount of medical items needed to treat a patient sick with cholera. Moreover, we reviewed the health facilities’ registries and clinical files to assess quantities and prices of items used to treat cholera. This was complemented by a thorough interview of involved health personnel to investigate the time spent by health personnel in treating cholera patients and the number of cholera cases treated in each facility. Costs incurred in setting-up a cholera treatment center (such as building and tent costs), under the general category of outbreak response-related costs and capital asset costs, were all excluded from this analysis. Diagnostic costs were not analyzed for the health facility study because of missing data. In addition, overhead costs were omitted in this analysis. The treatment cost of cholera to the health facility was estimated and further disaggregated into three levels of severity based on the length of hospital stay: 1) admitted for less than 12 hours (admitted <12 hours), 2) hospitalized for 1–3 days and 3) hospitalized for 4–7 days. The health facility study questionnaire is given in the supporting document **S2 Appendix**.

### Data management and analysis

Both household and provider databases were analyzed using Stata 13.1 (StataCorp, College Station, Texas, USA). All monetary values were presented in 2016 US dollar using the annual conversion rate between US dollar and Malawian Kwacha (MWK) of US$1 = MWK696.21. [26] To allow comparison between countries, we converted Malawian Kwacha to 2016 international dollars (I$) using the appropriate purchasing power parity conversion factor given by the International Monetary Fund. [27]

Regarding the data from the households’ interviews, a descriptive analysis was conducted to describe socioeconomic characteristics of patients and households and assess gender differences. The differences between proportions were tested using the chi squared test. The Mann–Whitney test was used to test for differences where distributions were skewed. The direct medical costs were estimated as the sum total of incurred costs before and during hospitalization of the patient while direct non-medical costs included only costs incurred during hospitalization. The indirect costs were only estimated for those who were working using the human capital approach. They were calculated based on self-reported total reduction of income induced by the cholera episode for patients and caregivers who lost income. The total cholera cost to a household was estimated as the sum of incurred costs, including direct out-of-pocket expenses in medicines and consumables before hospitalization, plus direct non-medical and indirect costs borne during hospitalization. The mean treatment (direct medical, non-medical, and indirect) cost to households was obtained by computing the average cost among patients. To assess the difference in mean cholera costs between groups of patients, we log-transformed the dependent variable, i.e., the mean cholera costs, to normalize the distribution. We then tested the difference of the mean cholera cost between groups of patients by running a log-linear regression of the mean cholera cost, adjusting for length of hospital stay. Given that poverty is widespread in the studied populations, coping strategies used to compensate for direct and indirect cholera costs may have huge impacts on households’ livelihood. We analyzed those coping strategies and the proportion of households that resorted to them to compensate for costs imposed by the disease.

The health facility cost of treating cholera by levels of case severity was obtained by adding-up costs of personnel, drugs and consumables. The estimated cost for each category of personnel was obtained by multiplying the estimated time spent treating a cholera case by their corresponding gross wage per minute. The total personnel cost was then obtained by adding-up these estimates. Likewise, the cost of each drug and consumable used in treating a patient was obtained by multiplying the units of each input by its price. The total cost on drugs and consumables was then calculated as the total sum spent on drugs and consumables according to levels of severity. Moreover, the average cost of treating an episode of cholera across groups of patients was reached by calculating the mean costs across the three categories of patients.

## Results

### Characteristics of patients and their caregivers

**Table 1** summarizes selected characteristics of the patients and their caregivers. All patients agreed to take part in the study. Males constituted 81% of the sample. The mean age for the patients was 27 years (range: 3–80). Women were not significantly older than their male counterparts (p = 0.7582). Ninety percent (90%) of the patients were hospitalized (for at least one day) and 5% died because of the disease. On average, patients were inactive, including for hospitalization, for up to 8 days. Women spent 7 days inactive compared to 9 days for men, but this difference was not statistically significant at the 95% confidence level (p = 0.9542). The inactive period duration for caregivers was 4 days. Fifty eight (58) patients and 72 caregivers were involved in income generating activities (result not showed). At the time of the interview, 92% of the participants had recovered and the remaining percentage was constituted with patients with missing information as to their status (3%).

**Table 1:** Selected characteristics of the study patients and their caregivers.

	All	Male	Female	P-value
**Sex**				
Frequency	100	81	19	
Percentage	100	81	19	NC
**Age**				
Mean (min-max)	27 (3–80)	27 (3–76)	28 (5–80)	0.7582
Standard deviation	14	13	16	
**Duration of illness episode, days**				
Mean	5	5	2	0.2848
Standard deviation	5	5	4	
**Length of patient inactive period, days**				
Mean	8	9	7	0.9542
Standard deviation	8	8	5	
**Length of caregiver inactive period, days**				
Mean	4	5	2	0.1049
Standard deviation	5	5	3	
**Distribution of patient across severity groups**				
Admitted <12 hours (%)	5	5	0	
Hospitalized (%)	90	71	19	
Deceased (%)	5	5	0	0.537
**Patient health status on interview date**				
Recovered (%)	92	73	19	
Deceased (%)	5	5	0	
Missing (%)	3	3	0	0.778

NC = Not calculated

### Mean treatment cost of cholera to household

On average, the treatment cost of cholera to patients’ households was US$65.6 in 2016 equivalent to I$249.9 (**Table 2**). Productivity losses (i.e., income losses due to days not worked) constituted almost 59% of the costs incurred by the patient’s household as a consequence of the cholera episode. Direct non-medical costs incurred in the facility came as the second most important cost driver, accounting for 40% of incurred costs. We were not surprised that drugs and consumables were the smallest cost driver, accounting for less than 1% of the costs incurred by the patient and the patient’s household for treating the disease given the national policy exempting patients from paying for cholera care.

**Table 2:** Mean cholera costs to the patient and his household in 2016 US$ and in I$.

	Cost		
	2016 US$	I$	Percentage
**Direct medical cost before visiting the facility**	**0.5**	**1.9**	**0.8**
Drugs and consumables	0.5	1.9	0.8
**Direct medical cost in the facility**	**0.0**	**0.1**	**0.1**
Drugs and consumables	0.0	0.1	0.1
**Direct non-medical cost in the facility**	**26.5**	**101,2**	**40.5**
Patient’s transportation	5.5	20.9	8.3
Caregivers’ transportation	3.1	11.7	4.7
Foods and beverages	15.8	60.1	24.1
Other miscellaneous	2.2	8.5	3.4
**Indirect (productivity losses)**	**38.5**	**146.7**	**58.6**
Patient	23.0	87.8	35.1
Caregivers	15.5	58.9	23.5
**Total cost**	**65.6**	**249.9**	**100.0**

### Average costs in various groups of patients

**Table 3** shows treatment costs of cholera across different groups of patients. Households of patients who died appeared to have incurred the largest treatment costs: US$106.5 (I$406.0) as compared to patients who were hospitalized US$51.9 (I$197.8) and those admitted for less than 12 hours US$38.3 (I$145.9) in 2016. However, this difference in mean costs was not statistically significant when controlling for the length of hospitalization (p = 0.4524). Productivity losses constituted the main cost for patients who had been hospitalized and those admitted for less than 12 hours, accounting for 72% and 95% of the costs incurred by these patients’ households, respectively. Because the survey instrument truncated productivity costs at death, a different pattern was observed for patients who died with indirect costs accounting for 39% of the total costs incurred in seeking care. Direct non-medical costs were the main cost driver, representing almost two-thirds of the total costs incurred by households of patients who died. Drugs and consumables represented only a marginal share (less than 2%) of the total costs incurred in seeking treatment regardless of patient category.

**Table 3:** Average treatment cost of cholera across groups of patients in 2016 US$ and I$.

	Admitted <12hours			Hospitalized			Deceased			
	2016US$	I$	%	2016US$	I$	%	2016US$	I$	%	P-value
**Direct medical cost before visiting the facility**	**0.3**	**1.1**	**0.7**	**0.7**	**2.8**	**1.4**	**0.5**	**1.9**	**0.5**	
Drugs and consumables	0.3	1.1	0.7	0.7	2.8	1.4	0.5	1.9	0.5	
**Direct medical cost in the facility**	-	**-**	-	**0.1**	**0.4**	**0.2**	-	**-**	-	
Drugs and consumables	-	-	-	0.1	0.4	0.2	-	-	-	
**Direct non-medical cost in the facility**	**1.8**	**6.8**	**4.7**	**13.5**	**51.4**	**26.0**	**64.3**	**245.3**	**60.4**	
Patient’s transportation	-	-	-	4.9	18.8	9.5	11.5	43.8	10.8	
Caregivers’ transportation	-	-	-	0.6	2.3	1.1	8.6	32.9	8.1	
Foods and beverages	1.8	6.8	4.7	6.8	25.8	13.1	38.8	147.8	36.4	
Other (batteries, etc,)	-	-	-	1.2	4.6	2.3	5.5	20.8	5.1	
**Indirect (productivity losses)**	**36.2**	**138.0**	**94.6**	**37.6**	**143.3**	**72.4**	**41.7**	**158.8**	**39.1**	
Patient	33.0	125.9	86.3	24.6	93.5	47.3	11.5	43.8	10.8	
Caregivers	3.2	12.0	8.3	13.0	49.7	25.1	30.2	115.0	28.3	
**Total cost**	**38.3**	**145.9**	**100.0**	**51.9**	**197.8**	**100.0**	**106.5**	**406.0**	**100.0**	0.4524^a^

a = Linear regression of log-transformed total cost of care on groups of patients; Missings are due to patients not able to recall data

### Economic consequences to households of a cholera episode

**Table 4** presents the consequences for households of having to deal with a cholera episode. More than one half of the sample of patients’ households (52%) reported difficulties in dealing with their episode of cholera. Furthermore, those who reported difficulties resorted to measures such as borrowing money (90%), selling household livestock (6%), crops (2%) or assets (2%) to compensate for cholera direct and indirect costs. Independently of case severity, borrowing money was the most common strategy used to compensate for cholera direct and indirect costs. However, the most severe coping strategies including disinvestment of livestock, crops or assets were solely used by hospitalized patients’ households.

**Table 4:** Distribution of coping strategies used by households to compensate for cholera direct and indirect costs.

		Admitted		
Used coping strategies to compensate	All	<12 hours	Deceased	Hospitalized
**for direct and indirect cholera costs**	**N (%)**	**N (%)**	**N (%)**	**N (%)**
Yes	52 (52)	3 (60)	1 (20)	48 (53)
No	48 (48)	2 (40)	4 (80)	42 (47)
**Type of coping strategy used**				
Borrowing money	47 (90)	3 (100)	1 (100)	43 (90)
Selling livestock	3 (6)	0 (0)	0 (0)	3 (6)
Selling crops	1 (2)	0 (0)	0 (0)	1 (2)
Selling assets	1 (2)	0 (0)	0 (0)	1 (2)

### Average treatment cost of cholera to the health centre

**Table 5** shows the average cost to the health centre for treating a cholera patient, and the distribution of costs across groups of patients. On average, the treatment cost of a patient with cholera was US$59.7 equivalent to I$227.5 in 2016. When disaggregating this mean cost according to the length of hospitalization, costs incurred by the health centre in treating patients who had been hospitalized for 4 to 7 days were US$125.0 (I$476.5) while US$50.7 (I$193.2) and US$3.4 (I$12.8) were incurred in 2016, respectively, for the treatment of patients who were hospitalized for 1 to 3 days and those who were admitted for less than 12 hours. Furthermore, personnel costs incurred in treating an episode of cholera progressed with the length of hospitalization. Among those admitted for less than 12 hours, 42.7% incurred costs were for personnel compared to a minimum of 84% for patients hospitalized for at least a day.

**Table 5:** Average treatment cost of cholera to the health centre in 2016 US$ and I$.

				Hospitalized			Hospitalized					
	Admitted <12 hours			1–3 days			4–7 days			Across groups		
	2016US$	I$	%	2016US$	I$	%	2016US$	I$	%	2016US$	I$	%
Personnel	1.5	5.5	42.7	42.4	161.6	83.6	116.6	444.5	93.3	53.5	203.9	89.6
Drugs, consumables	1.9	7.3	57.3	8.3	31.6	16.4	8.4	32.0	6.7	6.2	23.6	10.4
**Mean total**	**3.4**	**12.8**	**100.0**	**50.7**	**193.2**	**100.0**	**125.0**	**476.5**	**100.0**	**59.7**	**227.5**	**100.0**

## Discussion

This study estimated the cost associated with the treatment of cholera to health providers and patients and affected households in Machinga and Zomba. Our study sample was skewed towards adult men (mean age of 27 years) who had been hospitalized for the treatment of their illness, which may not represent the overall population of persons with cholera in the community. The mean duration of illness in our study was 5 days, in line with previous study findings which reported average illness durations falling between 4 to 7 days in Matlab (Bangladesh), Kolkata (India), North Jakarta (Indonesia), Beira (Mozambique), [28] and Zanzibar, (Tanzania) [29]. On average, durations of professional inactive period (8 days) were much longer than duration of illness (5 days). Out of the 97 patients with a known health status, 5 had died from the disease at the health facility. This translates to a case fatality ratio in our sample of 5%, well beyond the standard upper case fatality ratio limit of less than 1% if appropriate treatment is provided. [11] Patients included in this study were mostly cases that occurred at the beginning of the outbreak, and research has shown that the case fatality rate from cholera is especially high at the beginning of outbreaks. [30–32]

Patients sick with cholera and affected households incurred an economic loss of US$65.6 (I$249.9) in treating the episode of illness and in income reduction due to the cholera episode. Our cost estimate falls, broadly, between estimates reported by Poulos et al (2012) in their study of the cost of cholera in various study locations, including US$12.4 in Matlab (Bangladesh), US$17.9 in Kolkata (India), US$18.8 in Beira (Mozambique), and US$134 in North Jakarta (Indonesia) [28] after adjusting for inflation. Our study sample was skewed towards hospitalized persons while the four other study samples were more balanced in terms of gender and case severity. This may explain the higher cost estimates we found in our study. The results also showed that the treatment costs to patients and affected households varied by case severity. This is also in line with the study in Matlab (Bangladesh), Kolkata (India), Beira (Mozambique), and North Jakarta (Indonesia), which also found that costs increased with case severity [28]. Moreover, very little was spent on direct medical costs because the treatment was provided free of charge. This implies that early presentation for care and identification, and appropriate treatment may reduce the economic burden of cholera on households, and that access to care, including financial access is critical in this respect.

Indirect costs related to productivity loss were the primary contributor to household costs. Numerous studies have shown that indirect costs contribute substantially to costs borne by patients and affected households during an episode of cholera. [29,33] Productivity losses estimated the total reduction of income for patients and caregivers induced by the disease. Reporting the estimated productivity to days not worked, the estimated daily losses to the patient and caregiver were approximately US$2.88 and US$3.88, respectively. These incurred productivity losses were respectively more than two and three times the Malawian GDP per capita per day estimated at US$1.02 with the annual GDP per capita of US$372. [34] Additionally, 52% of the sample resorted to various coping strategies, including borrowing and selling assets or livestock to pay for cholera care costs and to deal with the consequences of lost income. Borrowing money or selling livestock may have long term economic and other consequences for households. [35]

Health facility costs from our study (US$ 59.7) fall within the range reported from previous studies, including US$61 in Zanzibar, Tanzania, in 2012 [29] and between US$17.6–28.4 in Matlab (Bangladesh), Kolkata (India), Beira in (Mozambique), and North Jakarta (Indonesia). [33] Similarly to what was found from the household survey analysis, health care costs increased with disease severity. At least 80% of the treatment costs for hospitalized patients and across groups of patients in general were personnel costs. Because cholera outbreaks are largely unpredictable, health care personnel are not hired specifically to deal with this illness but rather are pulled from other duties. This, in turn, has a cascading effect on care delivered for other illnesses, an effect we did not assess as it was out of our study scope.

## Limitations

This study had several limitations. First, our sample size was relatively small and thus may not be representative of the larger population in Lake Chilwa area. This was particularly the case when disaggregating cholera treatment costs by case severity. Second, the study did not collect household lost production linked to the illness. In addition, we did not value school days lost due to cholera. Because of this, indirect costs to households for treating cholera may have underestimated the true impact of cholera on economic income losses. [36] Third, five patients who suffered from cholera died. The analysis did not account for mortality costs so the household costs of fatal cholera cases were underestimated. Fourth, we did not include capital asset costs and other administrative and overhead costs. This also contributed to underestimating the treatment costs of cholera to the health facility. Moreover, the treatment costs of cholera to the health facility were underestimated because diagnostic cost data were unavailable and excluded from the analysis. Fifth, we considered only direct treatment costs and did not incorporate public health costs such as setting up and running cholera treatment centres, outbreak response and control, and surveillance systems. Because of this, the treatment costs of cholera to health centres may have been underestimated. Sixth, this study was conducted in specific fishermen populations located in the southern part of Malawi, who may not represent the broader population of Malawi. Given study perspectives, we did not incorporate numerous other costs, including reassigning health care personnel, loss of business investment and economic opportunities for affected communities, and loss of tourism. Seventh, cost data were collected retrospectively. This may have also contributed to distorting our cost estimates given possible recall biases, although minimized by the relatively shorter recall period. Eighth, we analyzed data from cholera cases independently of the vaccination status. Severity of cholera has been demonstrated to be lower among vaccinated people. [37] Because of this, the cost of illness estimates reported in this study may have been underestimated. Finally, our study took a retrospective data collection approach while Poulos et al [28] and Sarker et al [33] adopted a prospective approach with two set of interviews and a mixed of retrospective and prospective approach, respectively. This may explain some of the differences observed in costs estimates. In contrast, Schaetti et al [29] used a study methodology that was similar to the current study approach. This may explain similarities in the two study findings.

## Strengths

Despite the limitations we pointed, the study has a number of strengths. The published literature on economic evaluations comes mostly from Asian countries. With the increasing burden of cholera in sub-Saharan countries, studies conducted in Africa may be of critical importance to advocate for oral cholera vaccine use in various situations. Moreover, the demonstration of the relative high burden of cholera to health centres and patients’ households in Malawi, a country in which cholera is endemic, may be an important entry point in fostering more research on the burden of cholera in many other sub-Saharan countries affected by the disease. This study may therefore serve as a springboard for the development of more comprehensive study of the burden of cholera on economies at large.

## Conclusions

This study demonstrated the burden of cholera to health facilities, and patients and affected households of Machinga and Zomba, Malawi. The study findings suggest that preventing an episode of cholera may contribute to averting significant cholera-related costs to patients and households. It further shows that by averting cholera, health facilities may avert using scarce resources in treating cholera patients. Altogether, our findings have direct policy implications regarding investments in prevention of cholera.

## Supporting information

S1 Appendix Questionnaire for the cost-of-illness study of cholera to households. (DOCX)

S2 Appendix Questionnaire for the cost-of-illness study of cholera to the health facility. (DOCX)
