# Supplementary material for: Cost-of-illness of cholera to households and health facilities in rural Malawi
Source: PLoS One. 2017 Sep 21;12(9):e0185041. doi: 10.1371/journal.pone.0185041 (PMC5608291; doi:10.1371/journal.pone.0185041)
Supplement: S1 Appendix — (DOCX) [file pone.0185041.s001.docx]

**S1 Appendix: Questionnaire for the cost-of-illness study of cholera to households.**

| **For INTERVIEWER (to fill at the CTC/CTU where patient was identified)** |
| --- |
| **Interviewer’s Name:**  **Patient’s Name in the village:**  **Patient’s Father’s Name:**  **Patient’s sex:** ⬜ Male ⬜ Female  **Patient’s age:**  **Name and address of the CTC/CTU where patient was identified:** _________________________________________________________  **Date of patient’s admission (DD/MM/YYYY):** ⬜⬜/⬜⬜/⬜⬜⬜⬜  **Date of patient’s discharge (DD/MM/YYYY):** ⬜⬜/⬜⬜/⬜⬜⬜⬜  **Patient’s address:**  **Planned Date of Interview (DD/MM/YYYY):** ⬜⬜/⬜⬜/⬜⬜⬜⬜ |

| **Section 1. Introduction**  The questionnaire is aimed at an adult patient of 18 years or older who has no mental disease. If the patient is under 18 years old and/or has mental disease, an adult who was in charge of the patient during the cholera episode, or who know very well the expenditure on this cholera episode should answer the question for the patient. |
| --- |
| **Check the patient‘s identification** |
| - 1. What’s your name? (check the name with previous page)   2. What’s your (the patient’s) age? _______ years |
| - 1. Have you been in the [health facility’s name] during the last 4 weeks for cholera treatment?   ⬜ Yes ⬜ No 🡪stop interview   - 1. Do you have an identification card? ⬜ Yes; ⬜ No   (If yes, check the name, sex, age and address on the card with your registration. Otherwise, check the age and sex with previous page. If it does not fit, stop the interview. Otherwise continue the interview) |
| - 1. What’s your relationship to the patient?   ⬜ Self (patient) ⬜ Mother ⬜ Father  ⬜ Sister ⬜ Brother ⬜ Grandmother  ⬜ Grandfather ⬜ Neighbor  ⬜ Other (specify): |

| **Section 2. Patient’s household information**  We would like to know more about your family’s migration history, family members, and main income winners |
| --- |
| 2.1. Are you a migrant? ⬜ yes; ⬜ no (if no, go to question 2.4) |
| 2.2. If yes, when did you move to this region? (jj/mm/yyyy):  ⬜ ⬜/ ⬜ ⬜/ ⬜ ⬜ ⬜ ⬜ |
| 2.3. What’s the main reason of your migration? (Choose as many choices as needed)   - Flood - Landslide/mudslide - Earthquake - Volcanic eruption - Tsunami - Windstorm - Forest fire - Fire - Others |
| 2.4. How many persons are there in your household? ⬜⬜ persons, including ⬜⬜ kids (under 16 years old) ⬜⬜ adults (16 years or older) |
| 2.5. Which of the below family member generate income for the family ? (Choose as many choices as needed. Give the number of sisters and brothers that provide an income to the family in the parenthesis)  ⬜ Self (patient) ⬜ Mother ⬜ Father  ⬜ Sisters (number: ____ ) ⬜ Brothers (number: ____ ) ⬜ Grandmother  ⬜ Grandfather ⬜ Other (specify): |

| **Section 3. Patient’s income information**  We would like to know your (the patient’s) occupation and income level in normal time |
| --- |
| 3.1. During the week before the cholera episode, did the patient conduct any of these activities?   - Work for pay - Attend school - Housekeeping - Job searching - Other (specify______________________________________________)   (If choose “work for pay” then continue, otherwise jump to Section 4) |
| 3.2. Which category best describes the work you (the patient) did in your (patient’s) last job:   - Fishing - Casual worker in agriculture (other than fishing) - Casual worker not in agriculture - Unpaid family worker - Self-employed - Self-employed with unpaid family worker/temporary worker - Self-employed with permanent worker - Government worker - Private worker |
| 3.3. Was it a seasonal job?  ⬜ Yes (answer question 3.5); ⬜ No (answer question 3.4) |
| 3.4. What was your (the patient’s) average daily income when you (the patient) were (was) working at that job?   - No more than 100 Mkw per day - 101 ~ 200 Mkw per day - 201 ~ 300 Mkw per day - 301 ~ 400 Mkw per day - More than 400 Mkw per day |
| 3.5. If it was a seasonal job, what was the period of the work season? (specify period from which month to which month)  ____________ to ______________________  (if it was not a seasonal job, skip to question 3.8)  3.6. What is your monthly income during work season?   - No more than 100 Mkw per day - 101 ~ 200 Mkw per day - 201 ~ 300 Mkw per day - 301 ~ 400 Mkw per day - More than 400 Mkw per day   3.7. What is your monthly income out of work season?   - No more than 100 Mkw per day - 101 ~ 200 Mkw per day - 201 ~ 300 Mkw per day - 301 ~ 400 Mkw per day - More than 400 Mkw per day |
| 3.8. Was that a …? ⬜ wage; ⬜ Net profits (after taking out costs)  ⬜ Gross profits (including costs) |

| **Section 4. Care givers’ income information**  We would like to know the income information of the care giver(s) |
| --- |
| 4.1. When you (the patient) were sick due to cholera, who took care of you (the patient)? (multiple choices are possible. If several brothers and sisters took care of the patient, give the number in the parenthesis)  ⬜ 1 = Self (patient) ⬜ 2 = Mother ⬜ 3 = Father  ⬜ 4 = Sister (number: ) ⬜ 5 = Brother (number: )  ⬜ 6 = Grandmother ⬜ 7 = Grandfather ⬜ 8 = neighbor  ⬜ Other (specify): |
|  |
| For **each** of the care givers, we would like to know his/her general income information *( use as many questionnaires as the number of caregivers)* |
| ***Care giver 1.***  4.2. Relationship to the patient:  ⬜ Mother ⬜ Father ⬜ Sister ⬜ Brother  ⬜ Grandmother ⬜ Grandfather  ⬜ Other (specify): |
| 4.3. During the week before the cholera episode, did the care giver do any of these activities?   - Work for pay - Attend school - Housekeeping - Job searching - Other (specify______________________________________________)   (If choose “work for pay” then continue, otherwise jump to care giver 2) |
| 4.4. Which category best describes the work the care giver did in this job:   - Fishing - Casual worker in agriculture (other than fishing) - Casual worker not in agriculture - Unpaid family worker - Self-employed (including fish trader) - Self-employed with unpaid family worker/temporary worker - Self-employed with permanent worker - Government worker - Private worker |
| 4.5. Was it a seasonal job?  ⬜ Yes (answer question 4.7); ⬜ No (answer question 4.6)  4.6. What was the caregiver’s average daily income when you (the patient) were (was) working at that job?   - No more than 100 Mkw per day - 101 ~ 200 Mkw per day - 201 ~ 300 Mkw per day - 301 ~ 400 Mkw per day - More than 400 Mkw per day |
| 4.7. If it was a seasonal job, what was the period of the work season? (specify period from which month to which month)  ______________ to ______________________  (if it was not a seasonal job, skip to question 4.10)  4.8. What is your monthly income **during** work season?   - No more than 100 Mkw per day - 101 ~ 200 Mkw per day - 201 ~ 300 Mkw per day - 301 ~ 400 Mkw per day - More than 400 Mkw per day   4.9. What is your monthly income **out of** work season?   - No more than 100 Mkw per day - 101 ~ 200 Mkw per day - 201 ~ 300 Mkw per day - 301 ~ 400 Mkw per day - More than 400 Mkw per day |
| 4.10. Was that a …? ⬜ wage; ⬜ Net profits (after taking out costs) ;  ⬜ Gross profits (including costs) |

| ***Care giver 2.***  4.2. Relationship to the patient:  ⬜ Mother ⬜ Father ⬜ Sister ⬜ Brother  ⬜ Grandmother ⬜ Grandfather  ⬜ Other (specify): |
| --- |
| 4.3. During the week before the cholera episode, did the care giver do any of these activities?   - Work for pay - Attend school - Housekeeping - Job searching - Other (specify______________________________________________)   (If choose “work for pay” then continue, otherwise jump to care giver 2) |
| 4.4. Which category best describes the work the care giver did in this job:   - Fishing - Casual worker in agriculture (other than fishing) - Casual worker not in agriculture - Unpaid family worker - Self-employed (including fish trader) - Self-employed with unpaid family worker/temporary worker - Self-employed with permanent worker - Employed by the government - Employed by private company |
| 4.5. Was it a seasonal job?  ⬜ Yes (answer question 4.7); ⬜ No (answer question 4.6)  4.6. What was the caregiver’s average daily income when you (the patient) were (was) working at that job?   - No more than 100 Mkw per day - 101 ~ 200 Mkw per day - 201 ~ 300 Mkw per day - 301 ~ 400 Mkw per day - More than 400 Mkw per day |
| 4.7. If it was a seasonal job, what was the period of the work season? (specify period from which month to which month)  ______________ to ______________________  (if it was not a seasonal job, skip to question 4.10)  4.8. What is your monthly income **during** work season?   - No more than 100 Mkw per day - 101 ~ 200 Mkw per day - 201 ~ 300 Mkw per day - 301 ~ 400 Mkw per day - More than 400 Mkw per day   4.9. What is your monthly income **out of** work season?   - No more than 100 Mkw per day - 101 ~ 200 Mkw per day - 201 ~ 300 Mkw per day - 301 ~ 400 Mkw per day - More than 400 Mkw per day |
| 4.10. Was that a …? ⬜ wage; ⬜ Net profits (after taking out costs) ;  ⬜ Gross profits (including costs) |

| ***Care giver 2.***  4.2. Relationship to the patient:  ⬜ Mother ⬜ Father ⬜ Sister ⬜ Brother  ⬜ Grandmother ⬜ Grandfather  ⬜ Other (specify): |
| --- |
| 4.3. During the week before the cholera episode, did the care giver do any of these activities?   - Work for pay - Attend school - Housekeeping - Job searching - Other (specify______________________________________________)   (If choose “work for pay” then continue, otherwise jump to care giver 2) |
| 4.4. Which category best describes the work the care giver did in this job:   - Fishing - Casual worker in agriculture (other than fishing) - Casual worker not in agriculture - Unpaid family worker - Self-employed (including fish trader) - Self-employed with unpaid family worker/temporary worker - Self-employed with permanent worker - Employed by the government - Employed by the private company |
| 4.5. Was it a seasonal job?  ⬜ Yes (answer question 4.7); ⬜ No (answer question 4.6)  4.6. What was the caregiver’s average daily income when you (the patient) were (was) working at that job?   - No more than 100 Mkw per day - 101 ~ 200 Mkw per day - 201 ~ 300 Mkw per day - 301 ~ 400 Mkw per day - More than 400 Mkw per day |
| 4.7. If it was a seasonal job, what was the period of the work season? (specify period from which month to which month)  ______________ to ______________________  (if it was not a seasonal job, skip to question 4.10)  4.8. What is your monthly income **during** work season?   - No more than 100 Mkw per day - 101 ~ 200 Mkw per day - 201 ~ 300 Mkw per day - 301 ~ 400 Mkw per day - More than 400 Mkw per day   4.9. What is your monthly income **out of** work season?   - No more than 100 Mkw per day - 101 ~ 200 Mkw per day - 201 ~ 300 Mkw per day - 301 ~ 400 Mkw per day - More than 400 Mkw per day |
| 4.10. Was that a …? ⬜ wage; ⬜ Net profits (after taking out costs) ;  ⬜ Gross profits (including costs) |

| ***Care giver 3.***  4.2. Relationship to the patient:  ⬜ Mother ⬜ Father ⬜ Sister ⬜ Brother  ⬜ Grandmother ⬜ Grandfather  ⬜ Other (specify): |
| --- |
| 4.3. During the week before the cholera episode, did the care giver do any of these activities?   - Work for pay - Attend school - Housekeeping - Job searching - Other (specify______________________________________________)   (If choose “work for pay” then continue, otherwise jump to care giver 2) |
| 4.4. Which category best describes the work the care giver did in this job:   - Fishing - Casual worker in agriculture (other than fishing) - Casual worker not in agriculture - Unpaid family worker - Self-employed (including fish trader) - Self-employed with unpaid family worker/temporary worker - Self-employed with permanent worker - Employed by the government - Employed by the private company |
| 4.5. Was it a seasonal job?  ⬜ Yes (answer question 4.7); ⬜ No (answer question 4.6)  4.6. What was the caregiver’s average daily income when you (the patient) were (was) working at that job?   - No more than 100 Mkw per day - 101 ~ 200 Mkw per day - 201 ~ 300 Mkw per day - 301 ~ 400 Mkw per day - More than 400 Mkw per day |
| 4.7. If it was a seasonal job, what was the period of the work season? (specify period from which month to which month)  ______________ to ______________________  (if it was not a seasonal job, skip to question 4.10)  4.8. What is your monthly income **during** work season?   - No more than 100 Mkw per day - 101 ~ 200 Mkw per day - 201 ~ 300 Mkw per day - 301 ~ 400 Mkw per day - More than 400 Mkw per day   4.9. What is your monthly income **out of** work season?   - No more than 100 Mkw per day - 101 ~ 200 Mkw per day - 201 ~ 300 Mkw per day - 301 ~ 400 Mkw per day - More than 400 Mkw per day |
| 4.10. Was that a …? ⬜ wage; ⬜ Net profits (after taking out costs) ;  ⬜ Gross profits (including costs) |

| ***Care giver 4.***  4.2. Relationship to the patient:  ⬜ Mother ⬜ Father ⬜ Sister ⬜ Brother  ⬜ Grandmother ⬜ Grandfather  ⬜ Other (specify): |
| --- |
| 4.3. During the week before the cholera episode, did the care giver do any of these activities?   - Work for pay - Attend school - Housekeeping - Job searching - Other (specify______________________________________________)   (If choose “work for pay” then continue, otherwise jump to care giver 2) |
| 4.4. Which category best describes the work the care giver did in this job:   - Fishing - Casual worker in agriculture (other than fishing) - Casual worker not in agriculture - Unpaid family worker - Self-employed (including fish trader) - Self-employed with unpaid family worker/temporary worker - Self-employed with permanent worker - Employed by the government - Employed by private company |
| 4.5. Was it a seasonal job?  ⬜ Yes (answer question 4.7); ⬜ No (answer question 4.6)  4.6. What was the caregiver’s average daily income when you (the patient) were (was) working at that job?   - No more than 100 Mkw per day - 101 ~ 200 Mkw per day - 201 ~ 300 Mkw per day - 301 ~ 400 Mkw per day - More than 400 Mkw per day |
| 4.7. If it was a seasonal job, what was the period of the work season? (specify period from which month to which month)  ______________ to ______________________  (if it was not a seasonal job, skip to question 4.10)  4.8. What is your monthly income **during** work season?   - No more than 100 Mkw per day - 101 ~ 200 Mkw per day - 201 ~ 300 Mkw per day - 301 ~ 400 Mkw per day - More than 400 Mkw per day   4.9. What is your monthly income **out of** work season?   - No more than 100 Mkw per day - 101 ~ 200 Mkw per day - 201 ~ 300 Mkw per day - 301 ~ 400 Mkw per day - More than 400 Mkw per day |
| 4.10. Was that a …? ⬜ wage; ⬜ Net profits (after taking out costs) ;  ⬜ Gross profits (including costs) |

Use addtional questionnaire if more than 4 caregivers

| **Section 5. Patient’s cholera episode duration**  We would like to know who were involved in the cholera episode and how long did it last. | |
| --- | --- |
| 5.1. What’s your (the patient’s) current health situation? (If yes, answer question 5.2, otherwise skip to question 5.3)  ⬜ recovered from the cholera and in good health  ⬜ still sick with cholera  ⬜ no cholera symptom anymore but sick of other disease  ⬜ deceased  5.2. If still sick of cholera, how long has the cholera episode lasted so far (since the moment that you (the patient) had the first symptom, such as diarrhea, till now)? _____ days  5.3. If recovered from cholera, how many days have your (the patient’s) cholera episode lasted (from the moment that you (the patient) had the first symptom, such as diarrhea, until you (the patient) stopped treatment)? _____days | |
| 5.4. In total, how many days didn’t you (the patient) work due to cholera? _____days  5.5. According to you, how much you (the patient) would have earned during **the whole period** of cholera episode, if you (the patient) were not sick? ⬜⬜⬜,⬜⬜⬜,⬜⬜⬜.⬜⬜ Kwacha | |
| For each care giver, we would like to know the impact of cholera episode on his/her income  ***Care giver 1 (correspond to the care giver 1 in section 4)***  5.6a. How long in total (including the patient’s stay in hospital) did the care giver stay with the patient? ______________hours/days (choose one of these two units)  5.7a. According to you, how much the care giver 1 would have won if he/she had not stayed with the patient? ⬜⬜⬜,⬜⬜⬜,⬜⬜⬜.⬜⬜ Kwacha  ***Care giver 2 (correspond to the care giver 2 in section 4)***  5.6b. How long in total (including the patient’s stay in hospital) did the care giver stayed with the patient? ______________hours/days (choose one of these two units)  5.7b. According to you, how much the care giver 2 would have won if he/she had not stayed with the patient? ⬜⬜⬜,⬜⬜⬜,⬜⬜⬜.⬜⬜ Kwacha  ***Care giver 3 (correspond to the care giver 3 in section 4)***  5.6c. How long in total (including the patient’s stay in hospital) did the care giver stayed with the patient? ______________hours/days (choose one of these two units)  5.7c. According to you, how much the care giver 3 would have earned if he/she had not stayed with the patient? ⬜⬜⬜,⬜⬜⬜,⬜⬜⬜.⬜⬜ Kwacha  ***Care giver 4 (correspond to the care giver 4 in section 4)***  5.6d. How long in total (including the patient’s stay in hospital) did the care giver stayed with the patient? ______________hours/days (choose one of these two units)  5.7d. According to you, how much the care giver 4 would have earned if he/she had not stayed with the patient? ⬜⬜⬜,⬜⬜⬜,⬜⬜⬜.⬜⬜ Kwacha  (Ask the same questions to additional care giver, and not it on an additional paper. ) | |
| 5.8. We would like to know whether you have treated yourself for your cholera:  ⬜ Consumed over-the-counter modern medicines.    ⬜ Consumed traditional herbs or traditional medicines as treatment  ⬜ Other, specify_________________________ | - If yes, the approximate total cost to purchase that medicine ⬜⬜⬜,⬜⬜⬜,⬜⬜⬜.⬜⬜ Kwacha - Don’t know - If yes, the approximate total cost to purchase that medicine ⬜⬜⬜,⬜⬜⬜,⬜⬜⬜.⬜⬜ Kwacha - Don’t know - If yes, the approximate total cost to purchase that medicine ⬜⬜⬜,⬜⬜⬜,⬜⬜⬜.⬜⬜ Kwacha - Don’t know |
| **Section 6. Transportation between health facility and the patient’s living place**  6.1. In the past 4 weeks, what health facilities did you visit to seek cholera treatment? (multiple choice is allowed)   - Cholera Treatment Center (CTC) - Cholera Treatment Unit (CTU) - Public health facility (other than CTC, CTU) - Private health facility - Traditional health provider - Other (specify) - Don’t know   6.2. How many kilometers was each of visited health facilities to the place where you stayed when you got cholera (your house or your floating home)?   \|  \| Type of health facility (refer to the choice in question 6.1) \| Distance between the health facility \| \| --- \| --- \| --- \| \| Health facility 1 \|  \| ⬜ ___________________km;  ⬜ Don’t know \| \| Health facility 2 \|  \| ⬜ ___________________km;  ⬜ Don’t know \| \| Health facility 3 \|  \| ⬜ ___________________km;  ⬜ Don’t know \| \| Health facility 4 \|  \| ⬜ ___________________km;  ⬜ Don’t know \|   ***For health facility 1***  6.3. What modes of transportation did you use to go to (one way) health facility 1   \| Modes of transportation \| Whether you used it \| If yes, estimate your approximate expenditure (including fuel cost, one way trip, etc) on this transportation to get to the health facility (one way) \| If yes, estimate the travel time (one way) you spent on each mode of transportation to get to the health facility 1 (one way) \| \| --- \| --- \| --- \| --- \| \| Boats \| ⬜ yes; ⬜ no \| ⬜⬜⬜,⬜⬜⬜,⬜⬜⬜.⬜⬜ Kwacha  ⬜ Don’t know \| _______________hours \| \| Car \| ⬜ yes; ⬜ no \| ⬜⬜⬜,⬜⬜⬜,⬜⬜⬜.⬜⬜ Kwacha  ⬜ Don’t know \| _______________hours \| \| Taxi \| ⬜ yes; ⬜ no \| ⬜⬜⬜,⬜⬜⬜,⬜⬜⬜.⬜⬜ Kwacha  ⬜ Don’t know \| _______________hours \| \| Horse cart \| ⬜ yes; ⬜ no \| ⬜⬜⬜,⬜⬜⬜,⬜⬜⬜.⬜⬜ Kwacha  ⬜ Don’t know \| _______________hours \| \| Motorcycle \| ⬜ yes; ⬜ no \| ⬜⬜⬜,⬜⬜⬜,⬜⬜⬜.⬜⬜ Kwacha  ⬜ Don’t know \| _______________hours \| \| Bicycle \| ⬜ yes; ⬜ no \| ⬜⬜⬜,⬜⬜⬜,⬜⬜⬜.⬜⬜ Kwacha  ⬜ Don’t know \| _______________hours \| \| On foot/walking \| ⬜ yes; ⬜ no \| ⬜⬜⬜,⬜⬜⬜,⬜⬜⬜.⬜⬜ Kwacha  ⬜ Don’t know \| _______________hours \| \| Others, specify: \| ⬜ yes; ⬜ no \| ⬜⬜⬜,⬜⬜⬜,⬜⬜⬜.⬜⬜ Kwacha  ⬜ Don’t know \| _______________hours \|   6.4. How many return trips you took to this health facility during your cholera treatment? ____________________________ | |
| 6.5. How many persons accompanied you (the patient) to the health facility 1 or come to take care of you (the patient)? ________________ persons  **For EACH accompanying person or caregiver, ask question 6.5 to 6.*7***  ***Person 1.***  6.6. What’s his/her relationship to you (the patient)? (multiple choice is allowed)  ⬜ Mother ⬜ Father ⬜ Sister ⬜ Brother ⬜ Grandmother ⬜ Grandfather ⬜ Neighbor  ⬜ Other (specify):  6.7. What was the transportation fee for the person 1 (return trip)?  ⬜ ⬜⬜⬜,⬜⬜⬜,⬜⬜⬜.⬜⬜ Kwacha  ⬜ Don’t know  6.8. How many trips he/she did to accompany/visit you at health facility 1? ______________  ***Person 2.***  6.6. What’s his/her relationship to you (the patient)? (multiple choice is allowed)  ⬜ Mother ⬜ Father ⬜ Sister ⬜ Brother ⬜ Grandmother ⬜ Grandfather ⬜ Neighbor  ⬜ Other (specify):  6.7. What was the transportation fee for the person 2 (return trip)?  ⬜ ⬜⬜⬜,⬜⬜⬜,⬜⬜⬜.⬜⬜ Kwacha  ⬜ Don’t know  6.8. How many trips he/she did to accompany/visit you at health facility 1? ______________  ***Person 3.***  6.6. What’s his/her relationship to you (the patient)? (multiple choice is allowed)  ⬜ Mother ⬜ Father ⬜ Sister ⬜ Brother ⬜ Grandmother ⬜ Grandfather ⬜ Neighbor  ⬜ Other (specify):  6.7. What was the transportation fee for the person 3 (return trip)?  ⬜ ⬜⬜⬜,⬜⬜⬜,⬜⬜⬜.⬜⬜ Kwacha  ⬜ Don’t know  6.8. How many trips he/she did to accompany/visit you at health facility 1? ______________  ***Person 4.***  6.6. What’s his/her relationship to you (the patient)? (multiple choice is allowed)  ⬜ Mother ⬜ Father ⬜ Sister ⬜ Brother ⬜ Grandmother ⬜ Grandfather ⬜ Neighbor  ⬜ Other (specify):  6.7. What was the transportation fee for the person 4 (return trip)?  ⬜ ⬜⬜⬜,⬜⬜⬜,⬜⬜⬜.⬜⬜ Kwacha  ⬜ Don’t know  6.8. How many trips he/she did to accompany/visit you at health facility 1? ______________ | |

| ***For health facility 2***  6.3. What modes of transportation did you use to go to (one way) health facility 1   \| Modes of transportation \| Whether you used it \| If yes, estimate your approximate expenditure (including fuel cost, one way trip, etc) on this transportation to get to the health facility (one way) \| If yes, estimate the travel time (one way) you spent on each mode of transportation to get to the health facility 1 (one way) \| \| --- \| --- \| --- \| --- \| \| Boats \| ⬜ yes; ⬜ no \| ⬜⬜⬜,⬜⬜⬜,⬜⬜⬜.⬜⬜ Kwacha  ⬜ Don’t know \| _______________hours \| \| Car \| ⬜ yes; ⬜ no \| ⬜⬜⬜,⬜⬜⬜,⬜⬜⬜.⬜⬜ Kwacha  ⬜ Don’t know \| _______________hours \| \| Taxi \| ⬜ yes; ⬜ no \| ⬜⬜⬜,⬜⬜⬜,⬜⬜⬜.⬜⬜ Kwacha  ⬜ Don’t know \| _______________hours \| \| Horse cart \| ⬜ yes; ⬜ no \| ⬜⬜⬜,⬜⬜⬜,⬜⬜⬜.⬜⬜ Kwacha  ⬜ Don’t know \| _______________hours \| \| Motorcycle \| ⬜ yes; ⬜ no \| ⬜⬜⬜,⬜⬜⬜,⬜⬜⬜.⬜⬜ Kwacha  ⬜ Don’t know \| _______________hours \| \| Bicycle \| ⬜ yes; ⬜ no \| ⬜⬜⬜,⬜⬜⬜,⬜⬜⬜.⬜⬜ Kwacha  ⬜ Don’t know \| _______________hours \| \| On foot/walking \| ⬜ yes; ⬜ no \| ⬜⬜⬜,⬜⬜⬜,⬜⬜⬜.⬜⬜ Kwacha  ⬜ Don’t know \| _______________hours \| \| Others, specify: \| ⬜ yes; ⬜ no \| ⬜⬜⬜,⬜⬜⬜,⬜⬜⬜.⬜⬜ Kwacha  ⬜ Don’t know \| _______________hours \|   6.4. How many return trips you took to this health facility during your cholera treatment? ____________________________ |
| --- | --- | --- | --- | --- | --- | --- | --- | --- | --- | --- | --- | --- | --- | --- | --- | --- | --- | --- | --- | --- | --- | --- | --- | --- | --- | --- | --- | --- | --- | --- | --- | --- | --- | --- | --- | --- |
| 6.5. How many persons accompanied you (the patient) to the health facility 1 or come to take care of you (the patient)? ________________ persons  **For EACH accompanying person or caregiver, ask question 6.5 to 6.*7***  ***Person 1.***  6.6. What’s his/her relationship to you (the patient)? (multiple choice is allowed)  ⬜ Mother ⬜ Father ⬜ Sister ⬜ Brother ⬜ Grandmother ⬜ Grandfather ⬜ Neighbor  ⬜ Other (specify):  6.7. What was the transportation fee for the person 1 (return trip)?  ⬜ ⬜⬜⬜,⬜⬜⬜,⬜⬜⬜.⬜⬜ Kwacha  ⬜ Don’t know  6.8. How many trips he/she did to accompany/visit you at health facility 1? ______________  ***Person 2.***  6.6. What’s his/her relationship to you (the patient)? (multiple choice is allowed)  ⬜ Mother ⬜ Father ⬜ Sister ⬜ Brother ⬜ Grandmother ⬜ Grandfather ⬜ Neighbor  ⬜ Other (specify):  6.7. What was the transportation fee for the person 2 (return trip)?  ⬜ ⬜⬜⬜,⬜⬜⬜,⬜⬜⬜.⬜⬜ Kwacha  ⬜ Don’t know  6.8. How many trips he/she did to accompany/visit you at health facility 1? ______________  ***Person 3.***  6.6. What’s his/her relationship to you (the patient)? (multiple choice is allowed)  ⬜ Mother ⬜ Father ⬜ Sister ⬜ Brother ⬜ Grandmother ⬜ Grandfather ⬜ Neighbor  ⬜ Other (specify):  6.7. What was the transportation fee for the person 3 (return trip)?  ⬜ ⬜⬜⬜,⬜⬜⬜,⬜⬜⬜.⬜⬜ Kwacha  ⬜ Don’t know  6.8. How many trips he/she did to accompany/visit you at health facility 1? ______________  ***Person 4.***  6.6. What’s his/her relationship to you (the patient)? (multiple choice is allowed)  ⬜ Mother ⬜ Father ⬜ Sister ⬜ Brother ⬜ Grandmother ⬜ Grandfather ⬜ Neighbor  ⬜ Other (specify):  6.7. What was the transportation fee for the person 4 (return trip)?  ⬜ ⬜⬜⬜,⬜⬜⬜,⬜⬜⬜.⬜⬜ Kwacha  ⬜ Don’t know  6.8. How many trips he/she did to accompany/visit you at health facility 1? ______________ |

| ***For health facility 3***  6.3. What modes of transportation did you use to go to (one way) health facility 1   \| Modes of transportation \| Whether you used it \| If yes, estimate your approximate expenditure (including fuel cost, one way trip, etc) on this transportation to get to the health facility (one way) \| If yes, estimate the travel time (one way) you spent on each mode of transportation to get to the health facility 1 (one way) \| \| --- \| --- \| --- \| --- \| \| Boats \| ⬜ yes; ⬜ no \| ⬜⬜⬜,⬜⬜⬜,⬜⬜⬜.⬜⬜ Kwacha  ⬜ Don’t know \| _______________hours \| \| Car \| ⬜ yes; ⬜ no \| ⬜⬜⬜,⬜⬜⬜,⬜⬜⬜.⬜⬜ Kwacha  ⬜ Don’t know \| _______________hours \| \| Taxi \| ⬜ yes; ⬜ no \| ⬜⬜⬜,⬜⬜⬜,⬜⬜⬜.⬜⬜ Kwacha  ⬜ Don’t know \| _______________hours \| \| Horse cart \| ⬜ yes; ⬜ no \| ⬜⬜⬜,⬜⬜⬜,⬜⬜⬜.⬜⬜ Kwacha  ⬜ Don’t know \| _______________hours \| \| Motorcycle \| ⬜ yes; ⬜ no \| ⬜⬜⬜,⬜⬜⬜,⬜⬜⬜.⬜⬜ Kwacha  ⬜ Don’t know \| _______________hours \| \| Bicycle \| ⬜ yes; ⬜ no \| ⬜⬜⬜,⬜⬜⬜,⬜⬜⬜.⬜⬜ Kwacha  ⬜ Don’t know \| _______________hours \| \| On foot/walking \| ⬜ yes; ⬜ no \| ⬜⬜⬜,⬜⬜⬜,⬜⬜⬜.⬜⬜ Kwacha  ⬜ Don’t know \| _______________hours \| \| Others, specify: \| ⬜ yes; ⬜ no \| ⬜⬜⬜,⬜⬜⬜,⬜⬜⬜.⬜⬜ Kwacha  ⬜ Don’t know \| _______________hours \|   6.4. How many return trips you took to this health facility during your cholera treatment? ____________________________ |
| --- | --- | --- | --- | --- | --- | --- | --- | --- | --- | --- | --- | --- | --- | --- | --- | --- | --- | --- | --- | --- | --- | --- | --- | --- | --- | --- | --- | --- | --- | --- | --- | --- | --- | --- | --- | --- |
| 6.5. How many persons accompanied you (the patient) to the health facility 1 or come to take care of you (the patient)? ________________ persons  **For EACH accompanying person or caregiver, ask question 6.5 to 6.*7***  ***Person 1.***  6.6. What’s his/her relationship to you (the patient)? (multiple choice is allowed)  ⬜ Mother ⬜ Father ⬜ Sister ⬜ Brother ⬜ Grandmother ⬜ Grandfather ⬜ Neighbor  ⬜ Other (specify):  6.7. What was the transportation fee for the person 1 (return trip)?  ⬜ ⬜⬜⬜,⬜⬜⬜,⬜⬜⬜.⬜⬜ Kwacha  ⬜ Don’t know  6.8. How many trips he/she did to accompany/visit you at health facility 1? ______________  ***Person 2.***  6.6. What’s his/her relationship to you (the patient)? (multiple choice is allowed)  ⬜ Mother ⬜ Father ⬜ Sister ⬜ Brother ⬜ Grandmother ⬜ Grandfather ⬜ Neighbor  ⬜ Other (specify):  6.7. What was the transportation fee for the person 2 (return trip)?  ⬜ ⬜⬜⬜,⬜⬜⬜,⬜⬜⬜.⬜⬜ Kwacha  ⬜ Don’t know  6.8. How many trips he/she did to accompany/visit you at health facility 1? ______________  ***Person 3.***  6.6. What’s his/her relationship to you (the patient)? (multiple choice is allowed)  ⬜ Mother ⬜ Father ⬜ Sister ⬜ Brother ⬜ Grandmother ⬜ Grandfather ⬜ Neighbor  ⬜ Other (specify):  6.7. What was the transportation fee for the person 3 (return trip)?  ⬜ ⬜⬜⬜,⬜⬜⬜,⬜⬜⬜.⬜⬜ Kwacha  ⬜ Don’t know  6.8. How many trips he/she did to accompany/visit you at health facility 1? ______________  ***Person 4.***  6.6. What’s his/her relationship to you (the patient)? (multiple choice is allowed)  ⬜ Mother ⬜ Father ⬜ Sister ⬜ Brother ⬜ Grandmother ⬜ Grandfather ⬜ Neighbor  ⬜ Other (specify):  6.7. What was the transportation fee for the person 4 (return trip)?  ⬜ ⬜⬜⬜,⬜⬜⬜,⬜⬜⬜.⬜⬜ Kwacha  ⬜ Don’t know  6.8. How many trips he/she did to accompany/visit you at health facility 1? ______________ |

| **Section 7. Expenditure on cholera treatment**  In this section, we would like to know how much you (the patient) and your family paid to treat your (the patient’s) cholera disease |
| --- |
| ***For health facility 1***  7.1a. How long did you stay at the health facility 1?  ⬜ If less than one day, then estimate how many hours: _____________________hour  ⬜ If more than one day, then estimate how many days: ________________________days  7.2a. Who accompanied you during your stay at the health facility 1?  ⬜ No body ⬜ Mother ⬜ Father  ⬜ Sister ⬜ Brother ⬜ Grandmother  ⬜ Grandfather ⬜ Neighbor  ⬜ Other (specify):  7.3 a. How long did each accompanying person stay with you at the health facility?  *Accompanying person 1 (what’s his/her relationship to the patient? ______________________)* ______________days ___________hours  *Accompanying person 2 (what’s his/her relationship to the patient? ______________________)* ______________days ___________hours  *Accompanying person 3 (what’s his/her relationship to the patient? ______________________)* ______________days ___________hours  7.4a. What were the expenditures on the treatment or consultation including prescription, medications, outpatient, inpatient that may have been administered upon your (the patient’s) discharge from the health facility?   \| *Treatment items* \| *Treatment expenditure* \| \| --- \| --- \| \| Prescription \| ⬜ ⬜⬜⬜,⬜⬜⬜,⬜⬜⬜.⬜⬜ Kwacha  ⬜ Didn’t pay anything  ⬜ Didn’t know \| \| Medications \| ⬜ ⬜⬜⬜,⬜⬜⬜,⬜⬜⬜.⬜⬜ Kwacha  ⬜ Didn’t pay anything  ⬜ Didn’t know \| \| Tests \| ⬜ ⬜⬜⬜,⬜⬜⬜,⬜⬜⬜.⬜⬜Kwacha  ⬜ Didn’t pay anything  ⬜ Didn’t know \| \| Overnight stay at health facility \| ⬜ ⬜⬜⬜,⬜⬜⬜,⬜⬜⬜.⬜⬜ Kwacha  ⬜ Didn’t pay anything  ⬜ Didn’t know \| \| Other (specify: _______________________) \| ⬜ ⬜⬜⬜,⬜⬜⬜,⬜⬜⬜.⬜⬜ Kwacha  ⬜ Didn’t pay anything  ⬜ Didn’t know \|   7.5a. What were the other costs incurred during your (the patient’s) stay at the health facility 1 (including the expenditures of the patient and accompanying persons)?   \| Cost item \| Total expenditure \| \| --- \| --- \| \| Food \| ⬜ ⬜⬜⬜,⬜⬜⬜,⬜⬜⬜.⬜⬜ Kwacha  ⬜ Didn’t pay anything  ⬜ Didn’t know \| \| Water \| ⬜ ⬜⬜⬜,⬜⬜⬜,⬜⬜⬜.⬜⬜ Kwacha  ⬜ Didn’t pay anything  ⬜ Didn’t know \| \| Accommodation \| ⬜ ⬜⬜⬜,⬜⬜⬜,⬜⬜⬜.⬜⬜ Kwacha  ⬜ Didn’t pay anything  ⬜ Didn’t know \| \| Additional transports during the patient stay at health facility \| ⬜ ⬜⬜⬜,⬜⬜⬜,⬜⬜⬜.⬜⬜ Kwacha  ⬜ Didn’t pay anything  ⬜ Didn’t know \| \| Others (specify: _____________________________) \| ⬜ ⬜⬜⬜,⬜⬜⬜,⬜⬜⬜.⬜⬜ Kwacha  ⬜ Didn’t pay anything  ⬜ Didn’t know \|   ***For health facility 2***  7.1b. How long did you stay at the health facility 2?  _____________days ______________hours  7.2b. Who accompanied you during your stay at the health facility 2?  ⬜ No body ⬜ Mother ⬜ Father  ⬜ Sister ⬜ Brother ⬜ Grandmother  ⬜ Grandfather ⬜ Neighbor  ⬜ Other (specify):  7.3 b. How long did each accompanying person stayed with you at the health facility 2?  *Accompanying person 1 (what’s his/her relationship to the patient? ______________________)* ______________days ___________hours  *Accompanying person 2 (what’s his/her relationship to the patient? ______________________)* ______________days ___________hours  *Accompanying person 3 (what’s his/her relationship to the patient? ______________________)* ______________days ___________hours  7.4b. What were the expenditures on the treatment or consultation including prescription, medications, outpatient, inpatient that may have been administered upon your (the patient’s) discharge from the health facility?   \| *Treatment items* \| *Treatment expenditure* \| \| --- \| --- \| \| Diagnosis \| ⬜ ⬜⬜⬜,⬜⬜⬜,⬜⬜⬜.⬜⬜Kwacha  ⬜ Didn’t pay anything  ⬜ Didn’t know \| \| Medications \| ⬜ ⬜⬜⬜,⬜⬜⬜,⬜⬜⬜.⬜⬜ Kwacha  ⬜ Didn’t pay anything  ⬜ Didn’t know \| \| Tests \| ⬜ ⬜⬜⬜,⬜⬜⬜,⬜⬜⬜.⬜⬜ Kwacha  ⬜ Didn’t pay anything  ⬜ Didn’t know \| \| Overnight stay at health facility \| ⬜ ⬜⬜⬜,⬜⬜⬜,⬜⬜⬜.⬜⬜ Kwacha  ⬜ Didn’t pay anything  ⬜ Didn’t know \| \| Other (specify: _______________________) \| ⬜ ⬜⬜⬜,⬜⬜⬜,⬜⬜⬜.⬜⬜ Kwacha  ⬜ Didn’t pay anything  ⬜ Didn’t know \|   7.5 b. What were the other costs incurred during your (the patient’s) stay at the health facility 1 (including the expenditures of the patient and accompanying persons)?   \| Cost item \| Total expenditure \| \| --- \| --- \| \| Food \| ⬜ ⬜⬜⬜,⬜⬜⬜,⬜⬜⬜.⬜⬜ Kwacha  ⬜ Didn’t pay anything  ⬜ Didn’t know \| \| Water \| ⬜ ⬜⬜⬜,⬜⬜⬜,⬜⬜⬜.⬜⬜ Kwacha  ⬜ Didn’t pay anything  ⬜ Didn’t know \| \| Accommodation \| ⬜ ⬜⬜⬜,⬜⬜⬜,⬜⬜⬜.⬜⬜ Kwacha  ⬜ Didn’t pay anything  ⬜ Didn’t know \| \| Additional transports during the patient stay at health facility \| ⬜ ⬜⬜⬜,⬜⬜⬜,⬜⬜⬜.⬜⬜ Kwacha  ⬜ Didn’t pay anything  ⬜ Didn’t know \| \| Others (specify: _____________________________) \| ⬜ ⬜⬜⬜,⬜⬜⬜,⬜⬜⬜.⬜⬜ Kwacha  ⬜ Didn’t pay anything  ⬜ Didn’t know \|   ***For health facility 3***  7.1 c. How long did you stay at the health facility 1?  ⬜ If less than one day, then estimate how many hours: _____________________hour  ⬜ If more than one day, then estimate how many days: ________________________days  7.2 c. Who accompanied you during your stay at the health facility 1?  ⬜ No body ⬜ Mother ⬜ Father  ⬜ Sister ⬜ Brother ⬜ Grandmother  ⬜ Grandfather ⬜ Neighbor  ⬜ Other (specify):  7.3 c. How long did each accompanying person stayed with you at the health facility?  *Accompanying person 1 (what’s his/her relationship to the patient? ______________________)* ______________days ___________hours  *Accompanying person 2 (what’s his/her relationship to the patient? ______________________)* ______________days ___________hours  *Accompanying person 3 (what’s his/her relationship to the patient? ______________________)* ______________days ___________hours  7.4c. What were the expenditures on the treatment or consultation including prescription, medications, outpatient, inpatient that may have been administered upon your (the patient’s) discharge from the health facility?   \| *Treatment items* \| *Treatment expenditure* \| \| --- \| --- \| \| Prescription \| ⬜ ⬜⬜⬜,⬜⬜⬜,⬜⬜⬜.⬜⬜Kwacha  ⬜ Didn’t pay anything  ⬜ Didn’t know \| \| Medications \| ⬜ ⬜⬜⬜,⬜⬜⬜,⬜⬜⬜.⬜⬜ Kwacha  ⬜ Didn’t pay anything  ⬜ Didn’t know \| \| Tests \| ⬜ ⬜⬜⬜,⬜⬜⬜,⬜⬜⬜.⬜⬜ Kwacha  ⬜ Didn’t pay anything  ⬜ Didn’t know \| \| Overnight stay at health facility \| ⬜ ⬜⬜⬜,⬜⬜⬜,⬜⬜⬜.⬜⬜ Kwacha  ⬜ Didn’t pay anything  ⬜ Didn’t know \| \| Other (specify: _______________________) \| ⬜ ⬜⬜⬜,⬜⬜⬜,⬜⬜⬜.⬜⬜ Kwacha  ⬜ Didn’t pay anything  ⬜ Didn’t know \|   7.5 c. What were the other costs incurred during your (the patient’s) stay at the health facility 1 (including the expenditures of the patient and accompanying persons)?   \| Cost item \| Total expenditure \| \| --- \| --- \| \| Food \| ⬜ ⬜⬜⬜,⬜⬜⬜,⬜⬜⬜.⬜⬜ Kwacha  ⬜ Didn’t pay anything  ⬜ Didn’t know \| \| Water \| ⬜ ⬜⬜⬜,⬜⬜⬜,⬜⬜⬜.⬜⬜ Kwacha  ⬜ Didn’t pay anything  ⬜ Didn’t know \| \| Accommodation \| ⬜ ⬜⬜⬜,⬜⬜⬜,⬜⬜⬜.⬜⬜ Kwacha  ⬜ Didn’t pay anything  ⬜ Didn’t know \| \| Additional transports during the patient stay at health facility \| ⬜ ⬜⬜⬜,⬜⬜⬜,⬜⬜⬜.⬜⬜ Kwacha  ⬜ Didn’t pay anything  ⬜ Didn’t know \| \| Others (specify: _____________________________) \| ⬜ ⬜⬜⬜,⬜⬜⬜,⬜⬜⬜.⬜⬜ Kwacha  ⬜ Didn’t pay anything  ⬜ Didn’t know \|   7.5 Have you and your family borrowed money to cover the expenditure caused by cholera episode?  ⬜ yes; (if yes, answer question 7.6)  ⬜ no (if no, skip to question 7.8)  7.6. If yes, how much?  ⬜ ⬜⬜⬜,⬜⬜⬜,⬜⬜⬜.⬜⬜ Kwacha  ⬜ Didn’t pay anything  ⬜ Didn’t know  7.7. How long do you think will be able to pay off your debt? ________days/months (choose the unit)  7.8. Did your (or the patient’s) household have to sell any items in order to raise money to pay for the cholera treatment?  ⬜ yes; (if yes, answer question 7.9)  ⬜ no (if no, finish this section)  7.9. What was/were sold?  ⬜ Clothing ⬜ Livestock, specify:  ⬜ Radio ⬜ Furniture  ⬜ Other (specify): _________________________________________ |

| **Final check (for interviewer)**  Interviewer’s name: ________________________  Interview’s data: **(DD/MM/YYYY**): ⬜⬜/⬜⬜/⬜⬜⬜⬜ |
| --- |
| Have you been able to accomplish the interview? ⬜ yes; ⬜ no |
| If no, what was the reason?  ⬜ the respondent was not there;  ⬜ the respondent did not correspond to the person identified at the health facility  ⬜ the respondent refused to participate to the survey  ⬜ other reason (specify: __________________________________________________________________) |
| Interviewer’s signature :____________________________________ |
