# Supplementary material for: Cost-of-illness of cholera to households and health facilities in rural Malawi
Source: PLoS One. 2017 Sep 21;12(9):e0185041. doi: 10.1371/journal.pone.0185041 (PMC5608291; doi:10.1371/journal.pone.0185041)
Supplement: S2 Appendix — (DOCX) [file pone.0185041.s002.docx]

**S2 Appendix: Questionnaire for the cost-of-illness study of cholera to the health facility.**

**Name of the health facility:__________________________________**

**Covered population:________________________________________**

**Address:__________________________________________________**

**Date of the interview (dd/mm/yy):____________________________**

**Position of the interviewed persons:**

1. **___________________________________________________**
2. **__________________________________________________**
3. **___________________________________________________**
4. **___________________________________________________**

| **Section 1. Cholera treatment protocol**  **In this section, we would like to know the general policy on cholera treatment** |
| --- |
| 1. Do you have any official protocol document for cholera treatment   Yes No (Skip to question 4)  (If yes, try to get of this document. Just copy parts concerning cholera treatment and note the document’ |
| 1. Is this protocol applied in the clinic practice in this health facility   Yes, totally applied Partially applied No applied at all |
| 1. If the protocol is not totally applied, please describe the real practice in this health facility |
| 1. Does the patient need to pay any medicine or services at this health facility for cholera treatment?  \| Treatment items \| Does the patient need to pay it? \| What’s the unit price (precise the unit) in Kwacha for the patient? \| \| --- \| --- \| --- \| \| Diagnosis \| Yes 🡪  No  N \| _________Kwacha/______(unit) \| \| Prescription \| Yes 🡪  No  N \| _________Kwacha/______(unit) \| \| Drugs (oral rehydration salts) \| Yes 🡪  No  N \| _________Kwacha/______(unit) \| \| Overnight stay at health facility \| Yes 🡪  No  N \| _________Kwacha/______(unit) \| \| Other*, specify:  ________________________ \| Yes 🡪  No  N \| _________Kwacha/______(unit) \| \| Other*, specify:  ________________________ \| Yes 🡪  No  N \| _________Kwacha/______(unit) \|   *”Other” means other goods and services that health facilities use to treat OCV. |

| **Section 2. Resource for cholera treatment**  **In this section, we would like to know which and how many resource were used to treat cholera** |
| --- |
| - 1. For outpatient treatment, on average, which of the following health facility professionals participate and how much time they spend to treat one cholera case  \| Health professionals \| Whether participate to outpatient cholera treatment \| On average, how many persons are needed to treat ONE outpatient case \| On average, how many time they spend to treat ONE outpatient case? (at first choose unit, then give the time estimation) \| \| --- \| --- \| --- \| --- \| \| Physician \| Yes 🡪  No  N \|  \| _____min \|____hour \| \| Nurse \| Yes 🡪  No  N \|  \| _____min \|____hour \| \| Health surveillance assitant \| Yes 🡪  No  N \|  \| _____min \|____hour \| \| Laborattory test persons \| Yes 🡪  No  N \|  \| _____min \|____hour \| \| Other*, specify:  ________________________ \| Yes 🡪  No  N \|  \| _____min \|____hour \| \| Other*, specify:  ________________________ \| Yes 🡪  No  N \|  \| _____min \|____hour \|  - 1. What was the monthly salary of the above mentioned health professionals?  \| **Health professional** \| **Monthly salary / person** \| \| --- \| --- \| \| Physician \| __________________Kwacha \| \| Nurse \| __________________Kwacha \| \| Health surveillance assitant \| __________________Kwacha \| \| Laborattory test persons \| __________________Kwacha \| \| Other*, specify:  ________________________ \| __________________Kwacha \| \| Other*, specify:  ________________________ \| __________________Kwacha \|  - 1. What medical materials were used to treat one cholera outpatient case?  \| **Medical materials** \| **Quantity for one outpatient treatment. Please precise the unit** \| \| **Unit price (the same unit as defined in the column on the left side)** \|  \| \| --- \| --- \| --- \| --- \| --- \| \| **Unit** \| **Quantity per treatment** \| \| Oral rehydration salts \|  \|  \|  \| \| Intravenous fuilds and electrolytes \|  \|  \|  \| \| Doxyclin \|  \|  \|  \| \| Azytromycin \|  \|  \|  \| \| Stool sample test \|  \|  \|  \| \| Zinc supplementation \|  \|  \|  \| \| Crystal VC dipstick \|  \|  \|  \| \| Other*, specify:  ________________________ \|  \|  \|  \| \| Other*, specify:  ________________________ \|  \|  \|  \|  - 1. For inpatient treatment, on average, which of the following health professionals participate and how much time they spend to treat one cholera case  \| Health professionals \| Whether participate to inpatient cholera treatment \| On average, how many persons are needed to treat ONE inpatient case \| On average, how many time they spend to treat ONE inpatient case? (at first choose unit, then give the time estimation) \| \| --- \| --- \| --- \| --- \| \| Physician \| Yes 🡪  No  N \|  \| _____min \|____hour \| \| Nurse \| Yes 🡪  No  N \|  \| _____min \|____hour \| \| Health surveillance assitant \| Yes 🡪  No  N \|  \| _____min \|____hour \| \| Laborattory test persons \| Yes 🡪  No  N \|  \| _____min \|____hour \| \| Other*, specify:  ________________________ \| Yes 🡪  No  N \|  \| _____min \|____hour \| \| Other*, specify:  ________________________ \| Yes 🡪  No  N \|  \| _____min \|____hour \|  - 1. What medical materials were used to treat one cholera inpatient case?  \| **Medical materials** \| **Quantity for one outpatient treatment. Please precise the unit** \| \| **Unit price (the same unit as defined in the column on the left side)** \|  \| \| --- \| --- \| --- \| --- \| --- \| \| **Unit** \| **Quantity per treatment** \| \| Oral rehydration salts \|  \|  \|  \| \| Intravenous fuilds and electrolytes \|  \|  \|  \| \| Doxyclin \|  \|  \|  \| \| Azytromycin \|  \|  \|  \| \| Stool sample test \|  \|  \|  \| \| Zinc supplementation \|  \|  \|  \| \| Crystal VC dipstick \|  \|  \|  \| \| Other*, specify:  ________________________ \|  \|  \|  \| \| Other*, specify:  ________________________ \|  \|  \|  \| |

| **Section 3. Cholera treatment**  **In this section, we want to know the number of cholera cases that were treated and their the length of hospital stay** |
| --- |
| 3.1 How many cases were treated in this health facility in February?  Outpatient:_________________ cases    7 Inpatients:__________________ cases    Stayed 1-3 days at the health facility: ____________________ cases  Stayed 4-7 days at the health facility:_____________________ cases  Stayed more than 7 days at the health facility:______________ cases  7 Deaths:__________________ cases |
| 3.2 How many patients returned to the health facility after his/her first visit_____________  What were the reasons? (possible to note several reasons) |
